# The ‘dark bronchus’ sign: HRCT diagnosis of *Pneumocystis carinii* pneumonia

**DOI:** 10.4103/1817-1737.30359

**Published:** 2007

**Authors:** Poonam Yadav, Ashu Seith, Rita Sood

**Affiliations:** Department of Radiodiagnosis, All India Institute of Medical Sciences, New Delhi, India; * *Department of Medicine, All India Institute of Medical Sciences, New Delhi, India*

**Keywords:** High resolution computerized tomography, *Pneumocystis carinii*, pneumonia

## Abstract

We report the importance of the ‘dark bronchus’ sign in the diagnosis of uniform, diffuse ground glass opacification on high resolution computerized tomography (HRCT). This sign is useful to identify diffuse ground glass opacity on HRCT in cases of *Pneumocystis carinii* pneumonia who may present with a normal or equivocal chest radiograph in the early course of disease.

A 50-year-old human immunodeficiency virus (HIV) positive male was investigated for symptoms of fever, shortness of breath and nonproductive cough of 3 weeks' duration. There was no associated hemoptysis. His CD 4 cell count was 89/mm^3^. Chest radiograph (postero-anterior view) did not reveal any significant abnormality [Figure 1]. However, in view of his chest symptoms and high suspicion of associated chest infection, computed tomography (CT) of chest was done. High resolution CT (HRCT) was performed at 1 mm collimation and 20 mm interval from the lung apices to the diaphragm (130 mA and 120 kVp).

**Figure 1:**
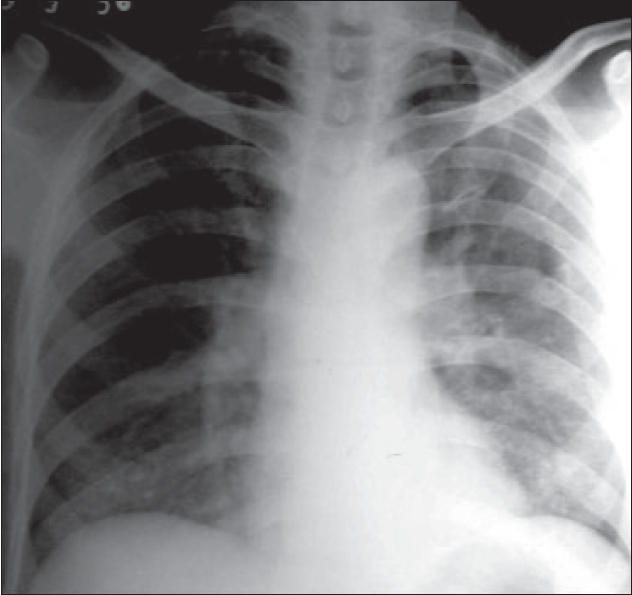
Chest radiograph (postero-anterior view) of the patient without any gross abnormality

There was no grossly evident abnormality on HRCT [Figure 2A]. Close inspection however revealed the ‘dark bronchus’ appearance [Figure 2B]. This sign provided the clue to the presence of generalized ground glass opacity. These findings were suggestive of *Pneumocystis carinii* pneumonia (PCP). The patient was put on therapeutic dose of Co-trimoxazole, which led to significant improvement in symptoms and resolution of fever. Repeat HRCT study after 2 weeks was found to be normal.

**Figure 2:**
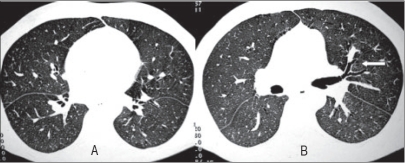
HRCT (axial section) showing presence of ‘dark bronchi’ relative to the surrounding lung parenchyma (arrow)

## Discussion

Chest radiograph is the initial investigation in HIV patients with chest symptoms. But even in patients with proven PCP, radiographic findings may be normal in up to 20-40%.[1,2] Low incidence of PCP in patients with normal or equivocal findings on chest radiograph despite high clinical suspicion emphasizes the need for a noninvasive and widely available investigation in such cases.

Various modalities to investigate symptomatic HIV patients with normal, equivocal or nonspecific radiographic findings include carbon monoxide diffusion in lung (DLCO), gallium citrate lung scanning and HRCT. A DLCO of less than 80% of the predicted value has a sensitivity of up to 98% for PCP,[3] but the specificity is less than 50% and the measurement is not always available. Although gallium scanning has a sensitivity of up to 100% for PCP in patients with abnormal radiographs,[4] it has never been prospectively studied in patients with normal or equivocal radiographic findings. In addition, this investigation requires a 48- to 72-hour delay in imaging, is not readily available and has a high cost.

On the other hand, HRCT is a widely available and noninvasive investigation for PCP. Patchy or diffuse ground glass opacity is the most frequent finding.[5] Other findings include cystic changes (33%), centrilobular nodules (25%), lymphadenopathy (25%) and pleural effusion (17%).[6] HRCT has been found to be especially important in the assessment of symptomatic patients with normal, equivocal or nonspecific radiographs. In such cases, it shows high sensitivity (100%), specificity (86%) and accuracy (90%) for PCP, using only the presence or absence of ground glass opacity as the criterion for positivity.[7]

Patchy ground opacity or mosaic attenuation, which is observed in up to 92% of the patients, can be easily identified on HRCT. However, subtle ground glass opacification, especially when bilateral and diffuse, may be difficult to diagnose. This is because of bilateral uniform increase in lung attenuation with absence of normal lung parenchyma for comparison. In such cases, the ‘dark bronchus’ appearance is a useful sign to identify diffuse ground glass opacity.[8] This finding refers to the presence of air-filled bronchi appearing ‘too black’ relative to the surrounding lung parenchyma, which is filled with inflammatory alveolar exudates. This subtle finding may help in identification of patients with ‘possible PCP’ despite a normal or equivocal chest radiograph. Subsequently direct test for PCP (i.e., broncho-alveolar lavage) may be initiated for definitive diagnosis and treatment.

We thus wish to emphasize the importance of the ‘dark bronchus’ sign in the diagnosis of uniform, diffuse ground glass opacification on HRCT. This is especially useful in the presence of a normal chest radiograph and ‘near normal’ HRCT. HRCT offers an accurate and early diagnosis in patients with normal chest radiographs; it alters patient management and facilitates early therapy.
